# Recent advances on visual cycle protein research and progress on clinical translation

**DOI:** 10.46439/ophthalmology.2.017

**Published:** 2020-09-08

**Authors:** Xin Yee Ooi, Rujman Khan, Anjalee Choudhury, Francisco Xavier Elisarraras, Jeff Grigsby, Brandi Obregon, Andrew Tsin

**Affiliations:** 1 Department of Molecular Science, University of Texas Rio Grande Valley School of Medicine, Edinburg, Texas, 78541, USA; 2 Baylor College of Medicine, Houston, Texas, 77030, USA; 3 Vision Health Specialties, Midland, Texas, 79707, USA; 4 Texas Tech University Health Science Center, Midland, Texas, 79705, USA

**Keywords:** Visual Cycle, DES1, IRBP, RPE 65, Gene Therapy, LCA, RP, DR, STGD

## Abstract

Since the publication of our previous paper, *Visual cycle proteins: Structure, function, and roles in human retinal disease* (Tsin, et.al, JBC 293:13016, 2018) there has been significant progress on multiple topics discussed in this paper. In the present communication, we further explore research advances on two visual cycle proteins: DES1 and IRBP. In addition, we emphasize the progress of clinical translation of other visual cycle protein research, including the breakthrough of FDA-approved gene therapy for Leber’s congenital amaurosis, and additional gene therapies at different stages of clinical trials for various retinal diseases such as retinitis pigmentosa, diabetic retinopathy, and Stargardt’s disease.

## Introduction

Since the publication of *Visual cycle proteins: Structure, function, and roles in human retinal disease*, there have been significant advances in research involving visual cycle proteins and the translation of basic science discoveries into treatment of retinal diseases, especially in terms of gene therapy. In our prior review article, specific visual cycle proteins, their gene mutations, and novel gene therapy treatments were summarized. Additional investigations, many of which involve clinical trials, have provided further information on the translational potential of visual cycle protein research to treat retinal diseases.

### Recent Advances in Visual Cycle Proteins Research

#### Interphotoreceptor Retinoid Binding Protein (IRBP)

As the most abundant soluble protein in the interphotoreceptor matrix (IPM), IRBP has been linked as a major contributor in maintaining the IPM’s integrity [1]. However, the effects of its presence and absence to visual structures requires further examination. Recently, researchers demonstrated the absence of IRBP through CRISPR knockout irbp-/- mice and the presence of IRBP in wildtype mice. In irbp-/-mice, the structure of the outer segments were abnormally altered, with thinning and change in space between the structures observed [2]. Because of the role of IRBP as a significant anti-oxidant in the retina, these results are consistent with others on IRBP-induced oxidative stress affecting the thickness of subretinal space [3,4].

A recent publication highlighted the significance of IRBP levels to combat detrimental effects of diabetes to vision. When examining vitreous and retina samples from healthy non-diabetic and diabetic patients, researchers discovered that there was a five-fold decrease in the expression of IRBP in patients with severe diabetic retinopathy (DR) in comparison to patients that did not have diabetes [5]. These researchers also established that there was a negative correlation between IRBP expression and severity of DR. Furthermore, a negative correlation between IRBP levels and levels of inflammatory cytokines (VEGF and IL-6) was also established. Thus, decreased IRBP levels may indicate a lack of resistance to the development of DR [5]. These novel findings reveal the opportunity to use IRBP as a biomarker when evaluating and treating DR.

IRBP is an important visual cycle protein to support visual function. In a recent study, HMG CoA reductase inhibitor (statin) simvastatin was found to reduce degeneration of rod photoreceptors and upregulate levels of IRBP under oxidative stress conditions induced by all-trans retinal [6]. Specifically, the study discovered that rod photoreceptors treated with simvastatin first had improved mitochondrial function when induced with oxidative stress. Further experiments are needed to investigate the detailed mechanism of simvastatin’s use in protecting photoreceptor degeneration through the increase of IRBP expression.

#### Isomerase enzyme of the intra-retinal visual cycle - DES1 and RGR

Dihydroceramide desaturase 1 (DES1) was considered the main enzyme involved in the photoisomerization reaction in the intraretinal/cone visual cycle, as it was thought to convert the retinoid chromophore back to *cis* configuration after the chromophore was converted to *trans* configuration in the photoisomerization reaction. It has been shown to convert all-*trans*-retinol to 11-*cis*-retinol [7]. However, recent studies using knockout DES1 retinas of mice and zebrafish show that the cone visual cycle has no interruptions, suggesting the DES1 does not have a significant role in supplying visual chromophore for cone pigment regeneration [8,9]. It has been reported that RGR (Retinal G-protein couple Receptor) opsin, working with retinol dehydrogenase 10, can provide 11-cis retinal for cone pigment regeneration in a light activated mechanism in Müller cells [10].

### Clinical Translation of Visual Cycle Protein Mutations - Gene Therapy for Retinal Disease

In addition to genetic research performed on mice models, human gene therapy has allowed for improved prognoses of previously irreversible blinding diseases such as retinitis pigmentosa, Leber’s congenital amaurosis, diabetic retinopathy, and Stargardt’s disease. The progression of gene therapy now exceeds the advancement of RPE65 gene treatment, with results promising a brighter future in vision.

#### Leber's congenital amaurosis (LCA)

While there are several mutations of visual cycle proteins associated with Leber’s congenital amaurosis (LCA), LCA caused by mutations of RPE65 is observed in nearly 6% of cases [11]. Focus on this pathology mechanism has grown rapidly because of the United States Food and Drug Administration’s 2017 approval of Luxturna (voretigene neparvovec-rzyl), a form of one-time gene therapy, to treat RPE65 mutations in LCA patients. Functional assays can be utilized to decide which specific RPE65 gene mutations are best suited for gene therapy in different individuals [12]. In one study, investigators used functional assays to determine the association between variants of uncertain significance (VUS) and their impact on RPE65 protein function as an indicator of gene therapy success. Measuring isomerase activity of RPE65 VUS using functional assays revealed some patients do not exhibit isomerase activity or proper protein function. Thus, making certain VUS unsuitable for gene therapy. In comparison, patients whose RPE65 VUS exhibited isomerase activity went on to receive successful gene therapy procedures [12]. Usage of functional assays allows for accurate estimation of VUS pathogenicity to determine gene therapy compatibility.

One of the markers of LCA is impairment of photoreceptors due to 11-cis-retinal deficiency and degradation of photoreceptor cells, often caused by proteasome stress [13]. Knockout mice models rpe65-/- (LCA2) and lrat -/- (LCA14) with deletion of M-opsins were used to determine the effects of opsin absence on photoreceptors. Mice with the deleted M-opsin gene experienced lower levels of proteasome stress and subsequent preservation of cone photoreceptors. Since cone opsins are found in higher quantities than other proteins, they have the largest impact on photoreceptor viability. However, investigators confirmed that cone photoreceptors were able to survive without cone opsins [13]. Through closer examination of this mechanism, there is potential in analyzing other options in addressing photoreceptor degeneration beyond 11-cis-retinal deficiency compensation. Further investigations may be conducted to pharmaceutically promote proteasome activity and photoreceptor preservation to supplement gene therapy.

The long term efficacy of gene therapy has been a looming yet present concern. Investigators recognized that RPE65 gene therapy was capable of retaining photoreceptors during primary onset of disease, before any degeneration of photoreceptors. However, gene therapy in later stages of the disease, when photoreceptor degeneration transpired, was substantially less effective. A group of researchers tested gene therapy effects at different stages of RPE65 gene therapy using animal models [14]. After initiating gene therapy in mutant canines with varying severity of photoreceptor loss and ages, researchers found that some regions in canine ocular tissue showed well-preserved photoreceptor nuclei in retinal regions with high expression of RPE65. Interestingly, the same research group found retinal regions where photoreceptors were viable with no RPE65 expression. Researchers noted that it was not the age of subjects, but the progression of the disease, primarily the extent of photoreceptor degeneration, that may determine long-term success of gene therapy [14]. With the current data in canines available, to translate the study to humans, it is important to consider that retinal degeneration may still occur even if disease conditions may be ameliorated.

There have also been new studies that investigate CRISPR gene-editing application for inherited retinal diseases. A subtype of LCA, LCA10, has had some recent gene editing advancements using in-vitro models. LCA10 involves a specific mutation in the ciliopathy gene *CEP290* [15,16]. Most LCA10 patients have the IVS26 mutation, which creates an erroneous splice in an intron sequence and results in the non-functional CEP290 protein. A potential candidate for LCA-type 10 treatment is EDIT-101 [16]. It uses an adeno-associated virus type 5 vector to transmit *S. Aureus* Cas9 and *CEP290*-specific guide RNAs (gRNAs) to photoreceptor cells via subretinal injection. In this study, researchers recognized two particular gRNAs that are specific to the *CEP290* gene locus, allowing for the gRNA to efficiently edit the mutated *CEP290* in human cells and retinal tissue. The pairing of SaCas9 and gRNAs to remove the inaccurate insertion of the intron sequence is shown to restore the *CEP290* gene for proper RNA splicing and consequent protein expression [16]. Other options in genetic alterations increase the chances of reversing this inherited retinal disease.

#### Retinitis pigmentosa (RP)

Previously, we indicated that retinitis pigmentosa (RP) presents as rod-cone dystrophy, meaning that there is a loss of rod photoreceptors before cone photoreceptors. As a result, recent studies focused on decreasing photoreceptor loss to improve visual function. The use of gene therapies, using adeno-associated vectors (AAV), AAV2/5-hPDE6β vector to target roc cGMP phosphodiesterase 6β are currently in phase 1 of clinical trials, with a high possibility to move on to the final stage of testing (phase 2) [17]. Other gene therapies currently in phase 1 of clinical trials are the AAV2/5-hRKp vector and the AAV vector RST-001 (Chop2), of which the latter is used to increase the photosensitivity gene to treat underlying conditions found in RP [17,18]. Additionally, current treatments under investigation include the same voretigene neparvovec-rzyl that was FDA-approved for LCA treatment, AAV serotype 8 (AAV8)-sCX3CL1, and ciliary neurotrophic factor (CNTF)- secreting neural stem cells (NSC) that have provided potential protection to photoreceptor cells in RP models [17–19]. The gene therapies currently under clinical trial demonstrate the promise of possible cures for RP.

Although RPE65 mutations account for a small percentage of RP cases, one of the major causes of X-linked RP includes a mutation on the RP GTPase (*RPGR*) gene that induces the thinning of the ONL. Since this gene is not as large as other genes that affect RP, *RPGR* has been the target of current research and development using AAV8 [20,21]. The idea of using AAV8 to transfect a codon-optimized *RPGR* coding sequence (AAV8-coRPGR) to treat *RPGR*-associated RP cases is currently being tested on human subjects [21]. Researchers established that patients receiving a higher dosage of the AAV8-coRPGR treatment showed a greater retention of improved visual field. Considerable progress has been made in establishing an alternative gene therapy to correct RP-related visual impairment, but more testing is needed to verify this prospective treatment.

#### Diabetic retinopathy (DR)

Previously, we mentioned some of the challenges in treatment of DR. Diabetic vascular complications remain one of the leading causes of blindness worldwide, and, although there have been significant improvements with the advent of anti-VEGF injections, it is still unclear what factors allow for preventing disease progression. It has been suggested that anti-VEGF therapies provide protection to Müller cells thus preventing further neural degradation [22]. Furthermore, researchers discovered that IRBP could block the damaging effects of VEGF and inflammatory cytokines by inhibiting tyrosine phosphorylation and by blocking GLUT1 to halt transportation of sugar into endothelial cells like Müller cells [5]. With this groundbreaking data, the study demonstrated that restoring IRBP levels up to physiologic baseline concentrations in DM rat models could potentially prevent or even reverse retinal abnormalities like vascular permeability [5]. In another study, diabetes-induced oxidative stress was attributable to superoxide from photoreceptors in early phase in the development of diabetic retinopathy [23]. Nonetheless, gene therapy investigations are underway to better understand the dynamic pathway leading to retinal neovascularization and vascular hyperpermeability.

Mesenchymal stem cell therapy has also demonstrated potential as immunomodulatory agents in DR, illustrating neuroprotective effects in light- and ischemia-induced retinal degeneration of animal models [17]. It is thought that stem cells exhibit neuroprotection by secreting neurotrophic factors, which promote tissue regeneration, inhibit fibrosis and apoptosis, and modulate the immune system to reduce inflammation [24]. In particular, endothelial progenitor cells (EPCs) and endothelial colony forming cells (ECFCs), a subtype of EPCs, have displayed promising capabilities of incorporating themselves into host retinal tissue and preventing dysfunctional angiogenesis [17,25,26]. Clinical trials have reported adequate safety and tolerance when evaluating stem cell treatment for photoreceptor degenerative diseases. However, future studies are needed to enhance knowledge of this type of therapy and to reinforce its efficacy [24,26].

#### Stargardt’s disease (STGD)

Stargardt’s disease (STGD) is caused by a number of mutations to the *ABCA4* gene [27]. Improper functioning of this transporter protein results in all-*trans*-retinaldehyde buildup, collapsing the visual cycle and leading to photoreceptor cell death. There is currently no effective therapy for the disease, but there is hope for a solution soon, as major progress has been in possible therapies. Direct gene replacement, AAV gene delivery, and lentiviral gene delivery have all been researched extensively, but lentiviral has been demonstrated to be most effective due to its capacity to securely carry *ABCA4* cDNA, which is nearly 7kb [28]. There are also non-viral gene therapies, mainly through nanoparticles, being researched.

Phase I/IIa clinical trials are being completed with lentiviral vectors and compacted DNA nanoparticules to treat patients [29]. A very recent publication using (1-aminoethyl)iminobis[N-(oleoylcysteinyl-1-amino-ethyl)propio-namide (ECO) to transduce wild-type *ABCA4* into mice shows great promise [30]. ECO is a pH-sensitive amino lipid that forms stable nanoparticles with nucleic acids; with pABCA4 plasmids, ECO easily forms stable nanoparticles. The ECO/pABCA4 nanoparticles self-assemble and have *in vitro* transfection efficiency. This system needs to be further modified to increase efficiency and enhance gene expression, but if successful, a therapy with little to no side-effects due to the nature of the nanoparticles could be used to treat STGD [30]. As *ABCA4* malfunction applies to STGD, types of RP, cone-rod dystrophy, and age-related macular degeneration, gene therapies to deliver functional *ABCA4* may also be useful in treating several other retinal diseases.

## Conclusion

The publication of *Visual cycle proteins: Structure, function, and roles in human retinal disease* highlighted the need to further explore pathways in the visual cycle and related clinical investigations including gene therapy. Recent discoveries about the role of IRBP, DES1, and RGR have further clarified the understanding of visual cycle pathway. Clinical trials are presently ongoing to test the effectiveness of gene therapy treatments. As additional research is conducted, our understanding of blinding diseases will be continual to expand leading to the likelihood of future breakthroughs to novel treatment of many ocular ailments.
